# Genome-wide analysis of *Jatropha curcas* MADS-box gene family and functional characterization of the *JcMADS40* gene in transgenic rice

**DOI:** 10.1186/s12864-020-6741-7

**Published:** 2020-04-28

**Authors:** Yuehui Tang, Jian Wang, Xinxin Bao, Qian Wu, Tongwen Yang, Han Li, Wenxia Wang, Yizhen Zhang, Nannan Bai, Yaxin Guan, Jiaxi Dai, Yanjie Xie, Shen Li, Rui Huo, Wei Cheng

**Affiliations:** 1 Key Laboratory of Plant Genetics and Molecular Breeding, Zhoukou Normal University, Henan, Zhoukou, China; 2 Henan Key Laboratory of Crop Molecular Breeding and Bioreactor, Henan, Zhoukou, China; 3 School of Journalism and Communication, Zhoukou Normal University, Henan, Zhoukou, China

**Keywords:** Physic nut, MADS-domain proteins, *JcMADS40*, Abiotic stress, Seed size, Transgenic plants

## Abstract

**Background:**

Physic nut (*Jatropha curcas*), an inedible oilseed plant, is among the most promising alternative energy sources because of its high oil content, rapid growth and extensive adaptability. Proteins encoded by MADS-box family genes are important transcription factors participated in regulating plant growth, seed development and responses to abiotic stress. However, there has been no in-depth research on the MADS-box genes and their roles in physic nut.

**Results:**

In our study, 63 *MADS-box* genes (*JcMADSs*) were identified in the physic nut genome, and classed into five groups (MIKC^C^, Mα, Mβ, Mγ, MIKC*) according to phylogenetic comparison with *Arabidopsis* homologs. Expression profile analysis based on RNA-seq suggested that many *JcMADS* genes had the strongest expression in seeds, and seven of them responded in leaves to at least one abiotic stressor (drought and/or salinity) at one or more time points. Transient expression analysis and a transactivation assay indicated that *JcMADS40* is a nucleus-localized transcriptional activator. Plants overexpressing *JcMADS40* did not show altered plant growth, but the overexpressing plants did exhibit reductions in grain size, grain length, grain width, 1000-seed weight and yield per plant. Further data on the reduced grain size in *JcMADS40*-overexpressing plants supported the putative role of *JcMADS* genes in seed development.

**Conclusions:**

This study will be useful in order to further understand the process of *MADS-box* genes involved in regulating growth and development in addition to their functions in abiotic stress resistance, and will eventually provide a theoretical basis for the functional investigation and the exploitation of candidate genes for the molecular improvement of physic nut.

## Background

The regulation of plant growth, development and stress responses is complex and is coordinated by many mechanisms. These mechanisms are under the control of many related genes acting through complex regulatory networks. In these processes, transcription factor (such as members of the MYB, HD-Zip, ARF, NAC, MADS-box, and ERF gene families) specifically recognize cis-regulatory elements present in the promoter regions of these genes, and regulate their expression so as to modulate a wide range of biochemical, physiological and developmental processes [1–6].

The MADS-domain proteins constitute one of the largest families of transcription factors. Structurally, almost all MADS-domain proteins contain a highly conserved DNA binding domain containing 58 amino acids residues, which can bind to DNA at consensus recognition sequences known as CC(A/T)6GG (CArG boxes), regulating the transcription of downstream genes [7]. Other domains are also present in the MADS-domain proteins; they include the keratin-like (K) domain, which is responsible for dimerization, the I (intervening) domain, which is capable of binding to form dimers, and the C (C-terminal) region, which is highly variable [8]. According to the similarities of amino acid sequences and structural features of the conserved domains, MADS-domain proteins in *Arabidopsis* can be classified into five groups (designated MIKC^C^, Mα, Mβ, Mγ, MIKC*) [9].

In recent years, many MADS-domain proteins have been widely identified and characterized, by means of genome-wide and expression profiles analysis researches, from a number of plant species including rice [10], wheat [11], potato [12], moso bamboo [13], *Arabidopsis* [9] and sheepgrass [14]. Studies on overexpression and mutant plants in dicots and monocots have suggested that members of MADS-box family play crucial roles in controlling plant growth and development [1]. For example, the *abs stk* double mutant has a reduced number of fertilized ovules and undergoes seed abortion resulting in very few seeds [15]. In rice, *OsMADS87* RNAi lines produce smaller seeds and overexpression (OE) lines have larger seeds compared with wild-type seeds [16]. The *Arabidopsis agl62* mutant shows accelerated endosperm cellularization, showing that *AGL62* plays an important regulatory role in inhibiting cellularization [17]. The *svp* and *flm* mutants exhibit temperature-insensitive flowering across wide temperature ranges [18], while *FUL* is necessary for leaf and fruit development and flowering in *Arabidopsis* [19]. *SEP1*, *SEP2*, and *SEP3* have been proven to be critical for the development of carpels, stamens, and petals [20]. In addition to participating in regulating plant growth, and development, evidence is accumulating to suggest that MADS-box genes also participated in plant responses to salt and drought stresses. For example, *CaMADS*-overexpressing *Arabidopsis* plants show increased resistance of salinity and cold stresses [21]. Loss-of-function mutations in *SVP* result in sensitivity to drought stress, whereas the *SVP*-overexpressing plants are more tolerant [22]. In tomato, *SlMBP11*-RNAi plants are less tolerance in response to salinity stress, but overexpressing this MADS-box gene confers salt stress tolerance [23]. However, although the functions of various members of MADS-box family have been made clear, little is known about the genes of this family and their roles in numerous taxa, containing the Euphorbiaceae.

Physic nut, a small woody member of the Euphorbiaceae, is an inedible oilseed plant grown mainly in tropical and subtropical regions and the oil in its seeds is used widely in industry [24]. Importantly, it has drawn much attention because it is one of the most suitable plants for producing biodiesel owing to its fast growth, ease reproduction, high oil content and wide adaptability [24]. Further research is thus necessary in order to clarify the molecular mechanisms of key genes in regulating physic nut development. The recent release of the physic nut genome sequence allows us to explore all the *JcMADS* genes at the genome level. However, there has as yet been no systematic study on the identities, expression patterns and functions of physic nut’s MADS-box genes in physic nut. To fill the deficiency, we firstly searched for and confirmed 63 MADS-box genes in physic nut (thereafter referred to as *JcMADS* genes). Secondly, we investigated their phylogenetic relationships, conserved motifs, chromosomal distribution, expression profiles and potential roles in physic nut development. Finally, *JcMADS40* was chosen for further functional analysis, and we tested its effects in transgenic rice. This study focuses on the functional roles of *JcMADS* genes in the development of physic nut.

## Results

### Identification of MADS-domain proteins in physic nut

All *Arabidopsis* MADS-domain protein sequences were used as queries in a BlastP search to identify physic nut proteins. A Hidden Markov Model (HMM) search was also performed against the protein database from physic nut by using the MADS-domain PF00319. In total, 63 putative MADS-domain proteins (designated JcMADS01 to JcMADS63) were identified in physic nut, with the presence of the MADS-domain in each of them being confirmed by a SMART website search. The open reading frame (ORF) lengths of 63 *JcMADS* genes ranged from 195 bp (*JcMADS35*) to 1164 bp (*JcMADS34*), thus potentially the proteins encoded would be from 64 to 387 amino acids; their GenBank accession numbers are given in Additional File 1.

### Phylogenetic relationships of the JcMADS proteins

**Fig. 1:**
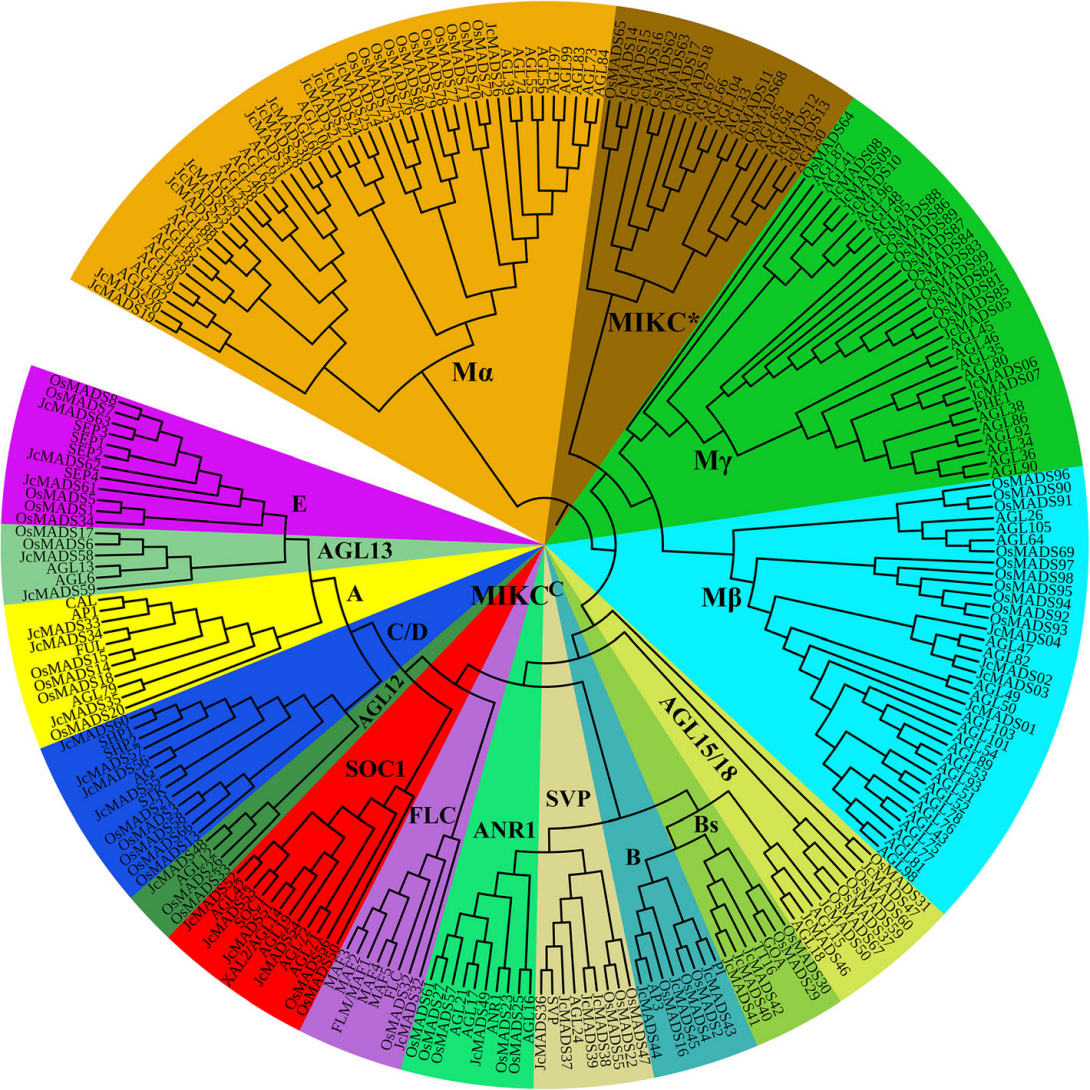
Unrooted phylogenetic tree of the MADS-box family proteins in physic nut, rice and *Arabidopsis*. The phylogenetic tree was constructed by IQ-TREE using the Maximum likelihood method

To clarify the phylogenetic relationships of the physic nut MADS family proteins with the previously reported members of the family in *Arabidopsis* and rice, an unrooted tree was constructed by IQ-TREE using the Maximum likelihood method (Fig. 1). On the basis of the similarity of full-length amino acid sequences, we subdivided the 246 typical members of the MADS gene family into 5 groups (designated MIKC^C^, Mα, Mβ, Mγ, MIKC*), according to the previous classification of MADS proteins from *Arabidopsis* [9, 25]. It was worth mentioning that the Mα, Mβ and Mγ belonged to type I, whereas the type II group contained MIKC* and MIKC^C^. Furthermore, the MIKC^C^ proteins, which could be further divided into 12 subfamilies (Fig. 1), based on the previous classification of MADS proteins [25]. Of the 63 inferred physic nut JcMADS proteins (Additional File 2), thirty-two were assigned to group MIKC^C^ (JcMADS32-JcMADS63), thirteen to group Mα (JcMADS19-JcMADS31), four to group Mβ (JcMADS01-JcMADS04), six to group Mγ (JcMADS05-JcMADS10) and eight to group MIKC* (JcMADS11-JcMADS18). In the phylogenetic tree, some members of the *JcMADS* gene family formed related sister pairs (Fig. 1): *JcMADS02* and *03*, *06* and *07*, *14* and *15*, *19* and *20*, *22* and *23*, *38* and *39*. There were also triplets (*JcMADS08*, *09* and *10*; *28*, *30* and *31*; *40*, *41* and *42*). The tree indicated that proteins in group MIKC^C^ were the most numerous; it contained 39 AtMADS, 38 OsMADS and 32 JcMADS proteins.

### Conserved motifs in JcMADS proteins

**Fig. 2:**
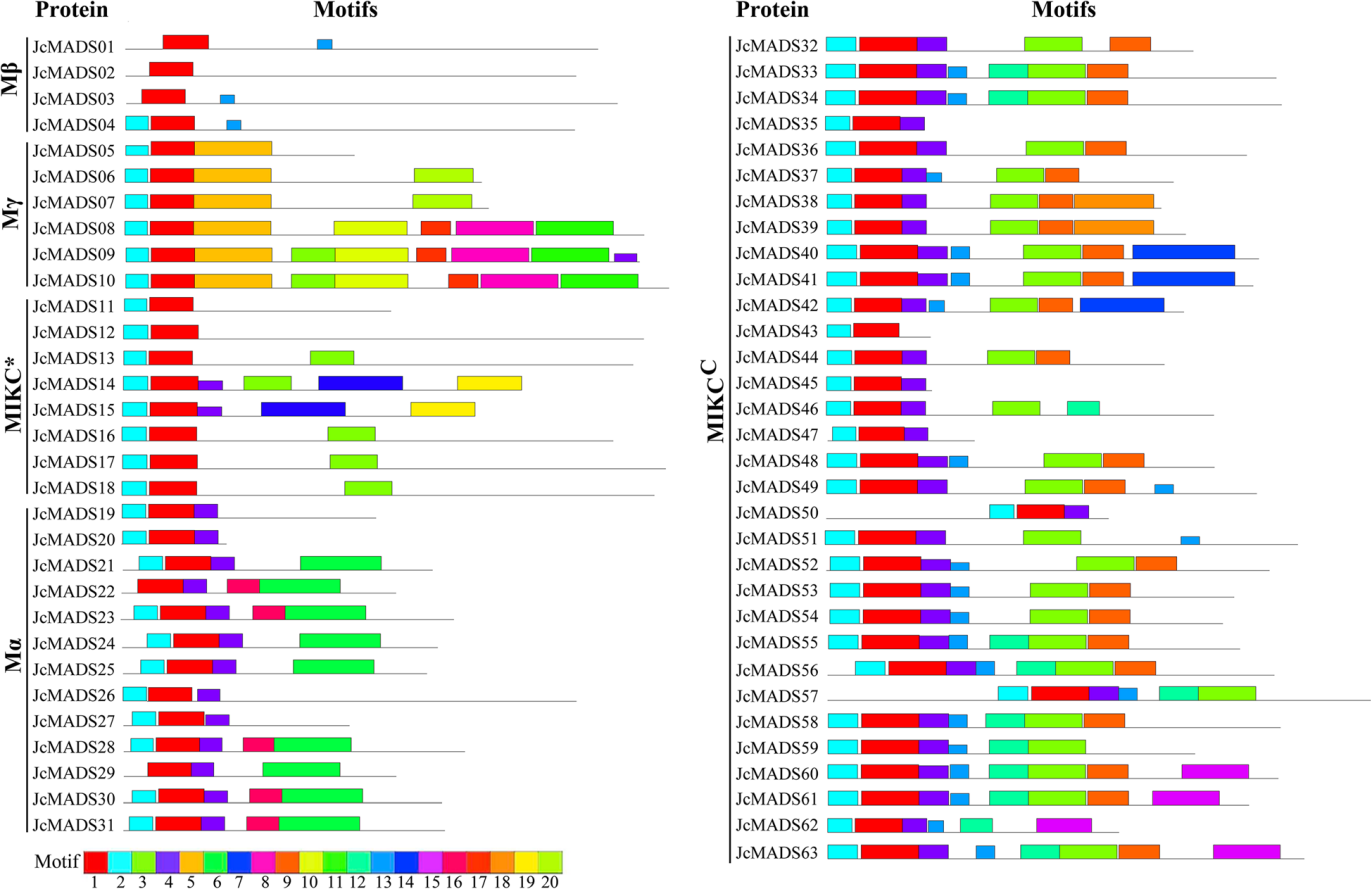
Conserved motifs within each MADS-domain protein in physic nut. Motifs were determined using MEME suite version 5.1.0. Non-conserved sequences were indicated by grey lines, and the colored box at the bottom represented the relative position of each conserved motif from each MADS-domain protein

The structures of proteins encoded by *JcMADS* genes were analyzed using the MEME online software tool. Twenty conserved motifs, which we named motifs 1–20, were identified in the 63 JcMADS proteins (Fig. 2 and Additional File 3). As expected, motif 1 and motif 4 corresponded to the typical MADS-domain, and motif 1 was found in all the JcMADS proteins. Motif 9, specifying the K domain, was found in most MIKC^C^ type proteins; the exceptions were JcMADS35, 43, 47, 50, 54 and 62, which have relatively short amino acid sequences. In addition to motifs with known functions, some motifs with unknown functions have also been detected. For example, motifs 5, 10 and 20 were observed only in group Mγ, whereas motif 13 was found only in groups MIKC^C^ and Mβ. Motif 12 was found only in group MIKC^C^, while motifs 14 and 19 was detected only in group MIKC*. Additionally, most conserved motifs detected in JcMADS proteins were clade-specifically assigned in different groups, suggesting similarity of function within a given group.

### Chromosomal localization of *JcMADS* genes

**Fig. 3:**
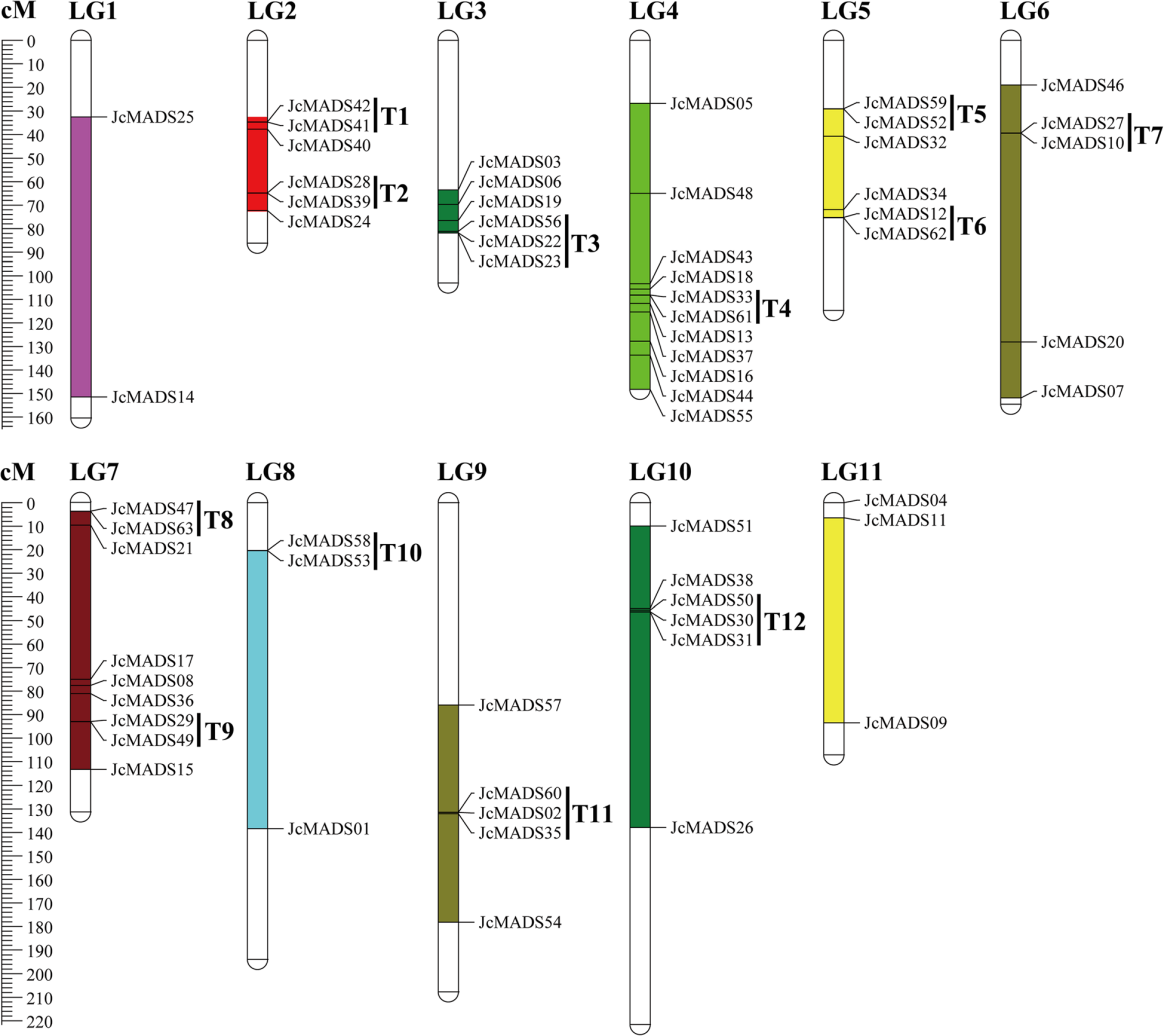
Chromosomal locations of physic nut *JcMADS* genes. In total, 62 *JcMADS* genes were located on 11 LGs (linkage groups). The top of each chromosome represents the number of chromosomes. cM (centiMorgans) shows the scale. T, tandem duplication

A total of 62 *JcMADS* genes, except *JcMADS45*, were mapped to linkage groups (LGs) based on a previously published report of physic nut [26]. As shown in Fig. 3, we found that LGs 4 and 7 had more members of the *JcMADS* gene family than other LGs, with eleven and nine *JcMADS* genes respectively. They were followed by LGs 2, 3, 5 and 10, each of which had six *JcMADS* genes. In addition, there was five *JcMADS* genes on each of LGs 6 and 9, three on LG8, three on LG11 and two on LG1. The results also indicated that most *JcMADS* genes were located in the lower and middle part from the LGs. Tandem duplications, defined as tandem repeats which are separated by < 4 non-homologous spacers or are genes located within 50 kb of each other [27], were found among these members of the *JcMADS* gene family. The gene pairs present as tandem repeats (T) were T1 (*JcMADS41* and *42*), T2 (*JcMADS28* and *39*), T3 (*JcMADS22*, *23* and *56*), T4 (*JcMADS33* and *61*), T5 (*JcMADS52* and *59*), T6 (*JcMADS12* and *62*), T7 (*JcMADS10* and *27*), T8 (*JcMADS47* and *63*), T9 (*JcMADS29* and *49*), T10 (*JcMADS53* and *58*), T11 (*JcMADS02*, *35* and *60*) and T12 (*JcMADS30*, *31* and *50*), on LG2, LG2, LG3, LG4, LG5, LG5, LG6, LG7, LG7, LG8, LG9 and LG10 respectively.

### Expression profile of *JcMADS* genes under non-stressed growth condition

**Fig. 4:**
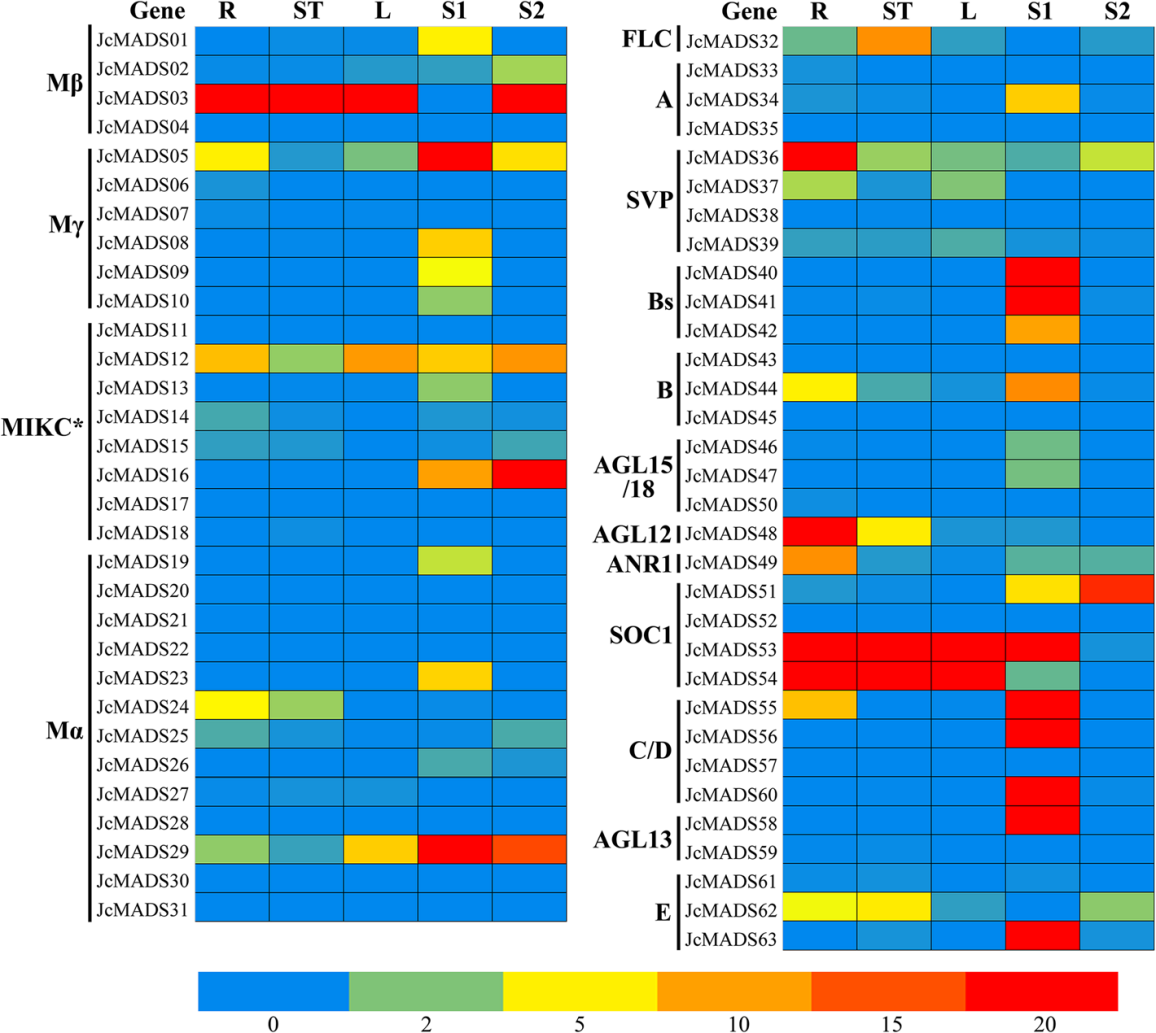
Patterns of expression of each *JcMADS* gene in physic nut roots (R), stem cortex (ST), leaves (L), S1(seeds at an early developmental stage) and S2 (seeds at filling stage), the levels of expression are displayed at the bottom by a colored scale

To clarify the roles of the *JcMADS* in regulating physic nut development, we examined the expression profiles of *JcMADS* genes in roots, stem cortex, leaves, and seeds (S1 and S2) under non-stressed growth conditions based on data from RNA sequencing (RNA-seq) (Additional File 4 and Fig. 4). The result suggested that fifty of the predicted *JcMADS* genes were expressed in at least one of the organs examined, while thirteen (*JcMADS04*, *11*, *17*, *20*, *21*, *22*, *28*, *30*, *31*, *35*, *43*, *45* and *57*) were not expressed in any of these tissues. Of the 50 *JcMADS* genes for which expression was detected, two (*JcMADS03* and *53*) were highly expressed across all the tissues sampled, ten (*JcMADS09*, *10*, *16*, *19*, *23*, *40*, *41*, *42,* 56 and *58*) were expressed only in seeds, thirteen (*JcMADS01*, *02*, *05*, *08*, *13*, *29*, *34*, *40*, *44*, *51*, *55*, *60*, and *63*) exhibited highest expression in seeds, four (*JcMADS24*, *36*, *48* and *49*) preferred to be expressed in roots, and one (*JcMADS32*) was most strongly expressed in the stem cortex.

Most *JcMADS* genes had higher expression in seeds at the S1 stage than at the S2 stage (Fig. 4). It was noteworthy that nine genes (*JcMADS01*, *09*, *10*, *23*, *40*, *42*, *55*, *56*, *48* and *58)* was examined expression only in seeds at S1 stage (Fig. 4). Based on the results of expression pattern analysis, the *JcMADS40* gene was chosen for functional research.

### Expression profile of *JcMADS* under abiotic stress conditions

**Fig. 5:**
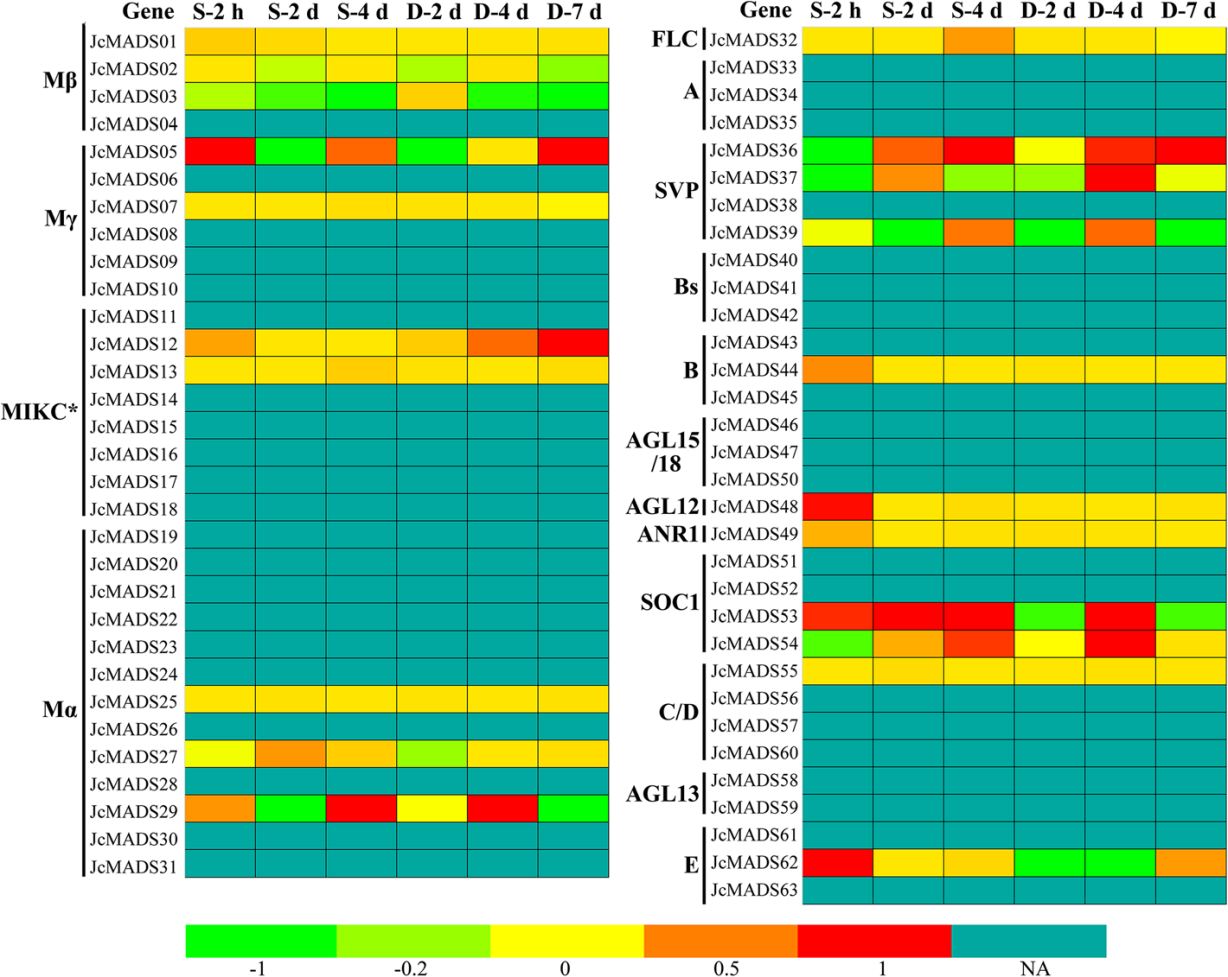
Levels of expression of the 63 *JcMADS* genes in physic nut leaves exposed to drought and salinity stresses: log_2_ ratios of signals from treated versus control leaves are presented as a heat map according to RNA-seq data, the color scale displayed at the bottom is used to display this value. Not available is indicated by NA

Many studies have suggested that some *MADS-box* genes encode proteins participated in the regulation of abiotic stresses [21, 28, 29]. We therefore further investigated the patterns of expression of *JcMADS* genes in leaves after 2 d, 4 d and 7 d of drought stress and after 2 h, 2 d and 4 d of salinity stress according to data from RNA-seq. Our data suggested that the transcript abundances of seven *JcMADS* genes indicated at least a twofold enhancement or reduction compared with the control in response to at least one stress at one or more time points (Fig. 5). Of these seven genes detected as having differential expression, three (*JcMADS29*, *36* and *53*) exhibited significantly induced or inhibited expression in response to drought and salinity stresses, three (*JcMADS12*, *37* and *54*) showed differential expression only in response to drought stress, and *JcMADS05* was solely affected by salt stress.

### *JcMADS40* gene encodes a transcriptional activator located in the nucleus

**Fig. 6:**
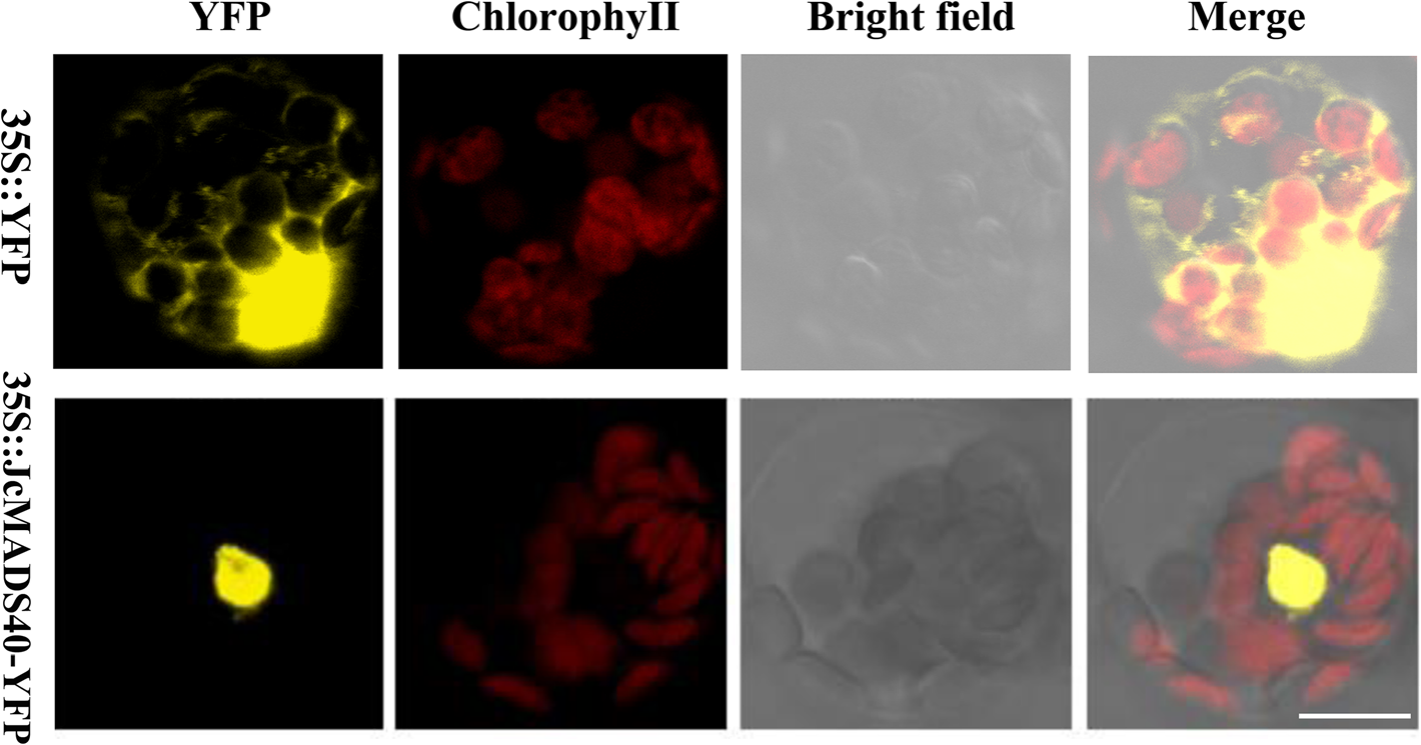
The product of the *JcMADS40* gene is localized in the nucleus. Scale bar, 10 μM

To confirm the subcellular localization of the protein encoded by *JcMADS40* gene, the 35S:JcMADS40-YFP fusion construct and the 35S:YFP empty vector were introduced into *Arabidopsis* protoplasts. The fluorescence signals from the protoplasts were then observed immediately by confocal laser-scanning microscopy. As shown in Fig. 6, we observed that the yellow fluorescent signal was distributed throughout the whole of the cell when the 35S:YFP vector was used, whereas in protoplasts harboring the construct 35S:JcMADS40-YFP a strong yellow fluorescent signal was detected in the nuclei. These findings indicate that *JcMADS40* gene is located in the nucleus.

**Fig. 7:**
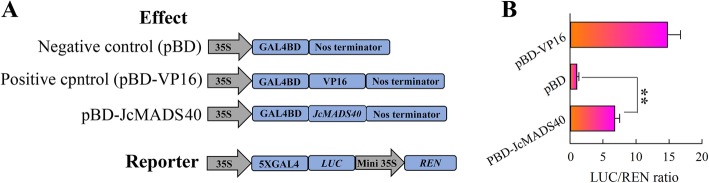
Transcriptional activity analysis of *JcMADS40* in *Arabidopsis* protoplasts. **a** Construction of reporter and effector vectors. **b** A dual-luciferase experiment indicated that *JcMADS40* functions as a transcription activator. Each assays included three biological replicates, and each contained two technical replicates (means of *n* = 6 ± SD; significant differences from controls (*p* < 0.01) are displayed with double asterisks above the bars)

A dual-luciferase assay was used to examine the transcription activation activity of JcMADS40 protein. The full-length CDS of *JcMADS40* was attached to the pBD vector, then the pBD-JcMADS40 fusion effector vector and the p5 × GAL-Reporter vector were transformed into *Arabidopsis* protoplasts. The results indicated that the LUC/REN ratio was obviously lower in the control protoplasts (pBD) than in the pBD-JcMADS40 group. Our data suggest that the full-length JcMADS40 has transactivation activity (Fig. 7). Based on the above results, we drew the conclusion that *JcMADS40* functions as a transcription activator.

### Phenotypic analysis of transgenic rice plants expressing *JcMADS40*

**Fig. 8:**
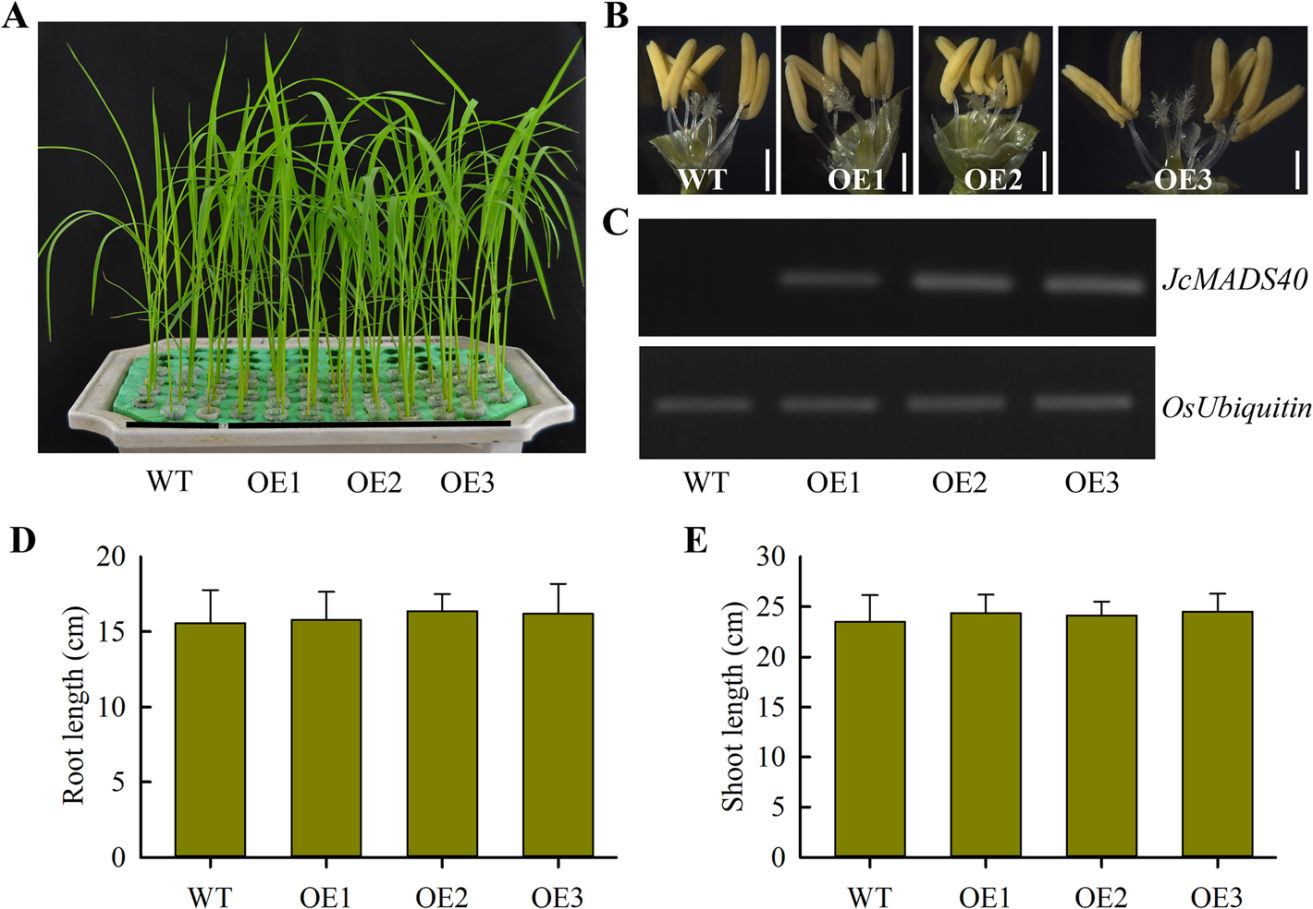
Characterization of *JcMADS40* overexpressed crops (OE1, OE2 and OE3) and their phenotypes. **a** Growth phenotype of two-week-old wild-type (WT) and *JcMADS40* overexpressed lines under exposure to non-stress conditions. **b** Flower structure in WT and overexpressed crops. **c** Levels of *JcMADS40* transcript in WT and overexpressed crops. **d** Root length in two-week-old WT and overexpressed crops. **e** Shoot length in two-week-old WT and overexpressed crops. Data presented in (**d**) and (**e**) are the means of *n* = 30 ± SD from three biological assays

To investigate the role of *JcMADS40* in regulating plant development, and to assess the feasibility of using *JcMADS* genes to control seed size in model crop, we further tested the effects of this gene in rice. Three independent transgenic lines (OE1, OE2 and OE3) were confirmed as expressing *JcMADS40* expression using semi-quantitative RT-PCR, and selected for further study. Expression of *JcMADS40* were detected in transgenic lines, whereas no expression was found in WT (wild-type) plants (Fig. 8c). Our results showed that the development and flower structure of transgenic plants overexpressing *JcMADS40* were very similar to those of WT lines (Fig. 8a and b). Statistical analysis indicated that there was no obvious difference in root and shoot lengths in the *JcMADS40* transgenic lines compared to the WT lines (Fig. 8d and e). Taken together, these results led to the conclusion that *JcMADS40* did not have any major effect on the growth of the *JcMADS40* transgenic plants.

### Overexpression of *JcMADS40* reduces the grain size in transgenic rice

**Fig. 9:**
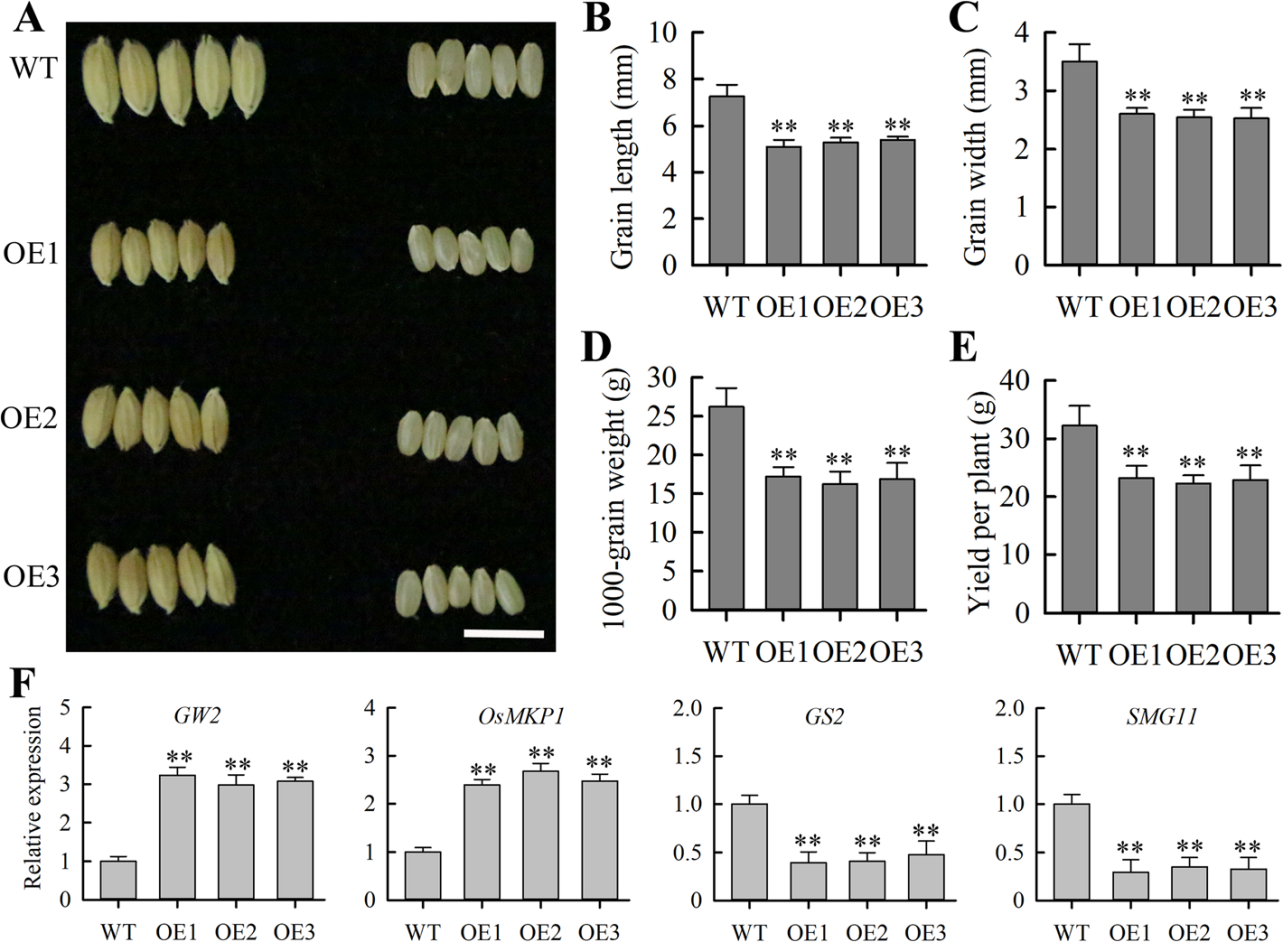
*JcMADS40* regulates grain size. **a** Grains from wild-type (WT) and *JcMADS40* overexpressed crops. Scale bar, 1 cm. **b** Grain length in WT and *JcMADS40* overexpressed crops. **c** Grain width in WT and *JcMADS40* overexpressed crops. **d** 1000-grain weight. **e** Yield per plant. All statistical results in **b**-**e** were calculated on plants grown at a spacing of 16 × 20 spacing in paddies under non-stress conditions. Data presented in (**b**), (**c**), (**d**) and (**e**) are the means of n = 30 ± SD (the double asterisks indicate *P* < 0.01 from the Duncan test). **f** Levels of seed-size-lated genes transcript. Each assay contained three biological replicates, and each contained two technical replicates (means of n = 6 ± SD; significant differences from controls (*p* < 0.01) are displayed with double asterisks above the bars)

As described above, *JcMADS40* expression was most strongly detected in seed, suggesting that *JcMADS40* might have significant roles in seed development. To verify this, we tested the effects of increasing *JcMADS40* expression on rice grain size. We found that *JcMADS40* transgenic plants produced dramatically smaller seeds than the WT lines (Fig. 9a). Our data also showed that *JcMADS40* overexpression plants had a significant reduction in both grain length and width compared to the WT plants (Fig. 9b and c). We also detected a significant reduction in 1000-seed weight, and yield per plant in *JcMADS40* transgenic lines (Fig. 9d and e). Our data suggested that overexpressing *JcMADS40* significantly altered seed size in transgenic plants.

To study the molecular mechanism of *JcMADS40* gene regulates grain size, we further tested the expression of grain-size-related genes (Fig. 9f). The results showed that expression of some positive regulatory factors, such as *GS2*, *SMG11*, was significantly lower in transgenic plants than that in wild-type, while expression of some negative regulatory factors, such as *OsMKP1*, *GW2*, was obviously higher than that in wild-type. Taken together, these data supported a putative role for *JcMADS* genes in seed development.

## Discussion

Increasing evidence suggests that members of the MADS-box family participate in a series of plant physiological phenomena. Up to now, many studies on the role of the MADS-box proteins are mainly concentrated in the model plants rice and *Arabidopsis* [27]. The molecular mechanisms of seed development in the biofuel plant physic nut, and more specifically the identities, expression patterns and functions of physic nut’s MADS-box proteins remain poorly understood. We therefore characterized and tested the expression patterns of MADS-box genes in physic nut, and chose one, *JcMADS40*, which was most strongly expressed in seed for further functional analysis by overexpressing this gene in rice.

In our research, a total of 63 MADS-box genes were confirmed in physic nut. It seems that the physic nut MADS-box family has a smaller number of members (genome size 320 Mb) compared to those in rice (genome size 466 Mb) and *Arabidopsis* (genome size 125 Mb) [25]. A possible reason for this fewer *JcMADS* genes is likely to be that MADS-box family members in the rice and *Arabidopsis* genome experience a chromosomal segment duplication event during the early evolution of these species [9, 10], whereas members of the physic nut MADS-box family do not experience such duplications [25]. Our phylogenetic tree showed that there were twenty-two *MADS* genes in the Mβ group in *Arabidopsis*, whereas there were only four *JcMADS* genes in group Mβ (Fig. 1). These finding suggests that the members of this group may have been either acquired in the *Arabidopsis* lineage or lost in the physic nut after divergence from the last common ancestor shared by *Arabidopsis* and physic nut. Motif analysis indicated that the distribution of motifs of the protein encoded by the *MADS* genes was diverse among different groups, whereas these members in the same group had a similar motif complement (Fig. 2), supporting their impregnable evolutionary conservation. Similar results have been observed in a variety of plants, including bread wheat [30], potato [12], moso bamboo [13], *Arabidopsis* [9] and sheepgrass [14]. Our results further show that the evolution and classification of the members of MADS-box family in physic nut are very conserved, as it is in other crops.

Preliminary predictions about the biological functions of genes and their products can be made by analyzing their expression profiles, we therefore detected the expression of 63 *MADS-box* genes sequencing-based transcriptome data. Our results show that *JcMADS55* expression was highest in seeds. Its *Arabidopsis* homolog *AGL11*(*STK*) is essential for seed development [15], and its homolog in oil palm *SHELL* controls oil production from seeds [31]. It can therefore be inferred from the high transcriptional abundance of *JcMADS55* in physic nut seeds that it may participated in regulation of the development of physic nut seed. *JcMADS48* had the highest transcription abundance in roots, and in *Arabidopsis,* its homologous *AGL12* is also preferentially expressed in root tissues and is essential for root development [32, 33]. The results indicated that *JcMADS48* may play an important role in physic nut root development. TT16, a MADS-box transcription factor, which affects seed development [34], and its homolog *JcMADS40* preferred to express in seeds, suggesting that *JcMADS40* may have a significant regulatory role in seed development. *SEPALLATA* (*SEP*) genes have been shown to have a key regulatory role in fleshy fruits development. Data related to fruit development and maturity are available in many plants, such as strawberry [35], bilberry [36], tomato [37], and others. The *SEP* homolog *JcMADS63*, was preferentially expressed in seeds, exhibiting that *JcMADS63* may play an important role in physic nut fruit development. *JcMADS03* and *53* were expressed in all tested physic nut tissues (Fig. 4), indicating that the functions of these genes may be throughout the development of physic nut plant. It is worth noting that many *JcMADS* genes showed preferential expression in seeds, implying that they may all be very important for physic nut plants in seed development. Overall, we deduce that *JcMADS* genes may have functions in each growing stages of physic nut plants; further research is required to confirm their roles.

Research increasingly have suggested that MADS-box genes participate in responses to various abiotic stresses in many crop species [21, 28, 29]. For example, *CaMADS*, which is strongly induced by salinity stress, and by ABA, acts as a mediator that has positive feedback effects in the process of pepper coping with cold, and salinity stress [21]. *OsMADS26* is a negative regulator in rice response to drought stress [29]. *AGL22* gene plays a crucial role in connecting changes in the initiation of drought stress and primary metabolism [38]. Although some researches have begun to screen certain *MADS-box* genes as important molecular components of drought and salinity stress responses, we have hitherto lacked complete information about the responses of members of MADS-box family to abiotic stresses (drought and salinity, etc.) in physic nut. In this study, RNA-based sequencing data in response to drought and salt stress, combined with qRT-PCR analysis, enabled us to screen *JcMADS* genes that respond to abiotic stress. For example, expression of *JcMADS29*, *36* and *53* was induced or inhibited by salt and drought stresses at one or more time points, while *JcMADS12*, *37* and *54* responded only to drought stress (Fig. 5). Collectively, we preliminary judgment these *JcMADS* genes may have significant roles in the regulation of abiotic stress responses in physic nut, and their functions merit further investigation.

Grain size is composed of grain width, grain length and grain thickness, which is an important agronomic trait that determines crop yield [39]. Although some genes involved in the regulation of seed size have been functionally studied in crop plants [40], the molecular mechanisms regulating rice seed size are still poorly understood. Additionally, *TT16* and *GOA*, two genes of the *Arabidopsis* B-sister group, have been cloned and functionally analyzed [33, 41, 42]. Previous studies have shown that *TT16* gene plays an important regulatory role in inner integument differentiation [41], and *GOA* plays a role as a negative regulatory factor in seed development [34, 42]. For example, increasing *GOA* expression significantly reduces seed size, on the contrary, decreasing the gene expression enhances seed size compared to wild-type [42]. In rape, *BnTT16* gene has been shown to be expressed mainly in seeds of early development, and the gene is necessary for seed development [43]. These findings together show that B-sister genes have an important regulatory role in seed development. In our work, we observed that a B-sister gene from the MIKC^C^ group, *JcMADS40*, had the highest expression in physic nut seeds (Fig. 4), and in order to verify its potential function, we further explored its roles in model crop rice. Rice as a monocot is more distantly related than physic nut and *Arabidopsis*, both Eudicots. Since B-sister function in rice and *Arabidopsis* is conserved [44], I agreed, it was valid to use rice. Therefore, in addition to demonstrating the function of the *JcMADS* genes, we also took this opportunity to verify the feasibility of using these genes to regulate seed development in rice crops. Our results showed that the *JcMADS40* transgenic plants had smaller, shorter and narrower grains and lower 1000-grain weight compared with wild-type plants (Fig. 9). Furthermore, our results also suggested that *JcMADS40* overexpressing plants reduced expression of *GS2* and *SMG11*, and increased expression of *OsMKP1* and *GW2* (Fig. 9f). *SMG11* overexpressing crops enhances grain size by up-regulating or down-regulating the transcriptional abundance of some grain-size-related genes [39]. Plants that increase *OsMKP1* expression have smaller grain compared to wild-type, conversely, results in larger grain [45]. *GW2* gene mutation enhances grain size and grain weight [46]. *GS2* acts as a positive regulator in the regulation of grain size and weight [46]. In short, *JcMADS40* overexpressing crops have smaller grains at least in part by affecting the transcription of these genes. These findings further support a role for *JcMADS40* in negative regulation of grain size. Reporter gene assays indicated that *JcMADS40* may function as a transcriptional activator, its role in negatively mediating grain size is likely to be regulated by the activation of other suppressors. In summary, the results can provide new gene resources for future explore the regulatory role of MADS-box family members in physic nut, especially with respect to their effects on seed size.

## Conclusions

In our research, we identified 63 *JcMADS* genes in physic nut, and characterized their expression profiles under normal growth and abiotic stress conditions. Transgenic plants that overexpress one of the members of the MADS-box family (*JcMADS40)* have a smaller grain, lower 1000-grain weight and yield per plant compared to wild-type plants, supporting this speculation that some *JcMADS* genes are involved in the regulation of seed development in physic nut. These findings can provide some valuable references in order to predict the function of MADS-box genes in stress tolerance and seed development, and comprehensive analysis of the gene family produced results that will be helpful in screening genes for further functional research and for the molecular improvement of yield traits in physic nut.

## Methods

### Plant materials

An inbred cultivar of *J. curcas*, GZQX0401, was used in this study owing to its genome sequencing has been completed [26]. The physic nut seeds in our research came from South China Botanical Garden, Chinese Academy of Sciences, Guangzhou, China. ZH11 (*japonica* cv. Zhonghua 11) was used as wild-type rice (*Oryza sativa* L.). Seed germination and cultivation of rice crops were carried out in pots filled with soil in the greenhouse of Zhoukou Normal University (China) under natural sunlight.

### Identification of MDAS-box gene in physic nut

One hundred and eight previously identified *Arabidopsis* MADS-domain protein sequences were used as queries in a search against the physic nut genome database. In addition, the HMM profile of the conserved MADS-domain (PF00319), obtained from the website of Pfam (Pfam 32.0, http://pfam.xfam.org/), was employed to carry out a BLASTP search against the physic nut database using the e-value (expected value) cut-off set at 0.01. All possible proteins identified as containing the MADS domain were confirmed through the SMART service (http://smart.embl-heidelberg.de/). The theoretical pI and molecular weight of all confirmed JcMADS proteins were determined using the ExPASy ProtParam tool (http://expasy.org/).

### Phylogenetic analysis

MADS protein sequences from *Arabidopsis* were downloaded from the TAIR database (https://www.arabidopsis.org/), and sequences for rice and *Jatropha curcas* were downloaded from GenBank (http://www.ncbi.nlm.nih.gov/). The ClustalX (1.83) program was employed to carry out alignment of multiple amino acid sequences. Phylogenetic trees comparing physic nut, rice and *Arabidopsis* MADS proteins were constructed using the Maximum likelihood method according to the similarity of full-length amino acid sequence, and the results were obtained with the IQ-TREE [47].

### Conserved motif and chromosomal distribution

The conserved motifs of individual MADS-domain proteins were identified using the MEME server (http://alternate.meme-suite.org/). MEME was performed according to the following requirements: site distribution (Zero or one occurrence per sequence), motif number (20), motif width (between 6 and 100 wide). Chromosomal locations of JcMADS proteins were obtained as described by Wu [26], and MapChart software package was employed to draw the linkage maps of JcMADS proteins.

### Expression profile analysis of *JcMADS* genes

Roots, stems and leaves of three-week-old physic nut seedlings, and seeds of 14 and 35 days after pollination from the same plants, were preserved and employed for further qRT-PCR and RNA-seq analysis. Three-week-old physic nut seedlings (six-leaf stage) were used for salt and drought stress treatments. The specific operations were as follows, the seedlings were directly irrigated with Hoagland solution containing 100 mM NaCl for salt stress, whereas watering was stopped for drought stress. And then leaves 2 d, 4 d, and 7 d after drought stress, and 2 h, 2 d, and 4 d after salt stress were preserved for subsequent analysis. Raw sequence data were obtained according to standard protocols, and uploaded to the SRA (sequence read archive) at NCBI (accession nos. Are PRJNA257901 (for the drought stress data) and PRJNA244896 (for the salt stress data)). The Illumina gene expression sample preparation kit was employed to prepare tag libraries of all RNA samples, and the Shenzhen BGI’s Illumina GAII platform was used for sequencing analysis. Next, the number of expressed tags was counted, then it was normalized to number of transcripts per million tags (TPM), and finally the level of gene transcript was determined. IDEG6 (http://telethon.bio.unipd.it/bioinfo/IDEG6_form/index.html) was employed to identify differentially expressed genes based on previous tests, applying the following requirements: the significance threshold was 0.01, and Bonferroni Correction [48]. Regarding the calculation method of salt and drought stress, the fold change of gene expression (abiotic stress/control) was twice or more was considered to be up-regulated or down-regulated.

### Subcellular localization and transcriptional activation analysis

The amplified coding region of *JcMADS40* without the termination codon was inserted into this vector (pSAT6-eYFP-N1) to generate 35S::JcMADS40-YFP. The 35S::JcMADS40-YFP fusion expression construct and the 35S::YFP empty vector were transferred into *Arabidopsis* protoplasts using the polyethylene glycol-mediated method. Subcellular localization of the control YFP and JcMADS40-YFP fusion proteins was observed under a confocal laser scanning microscope. *Arabidopsis* protoplasts were obtained following Tang [5].

For the transactivation assay of *JcMADS40*, the full-length *JcMADS40* gene was fused to the pBD vector to generate the construct pBD-JcMADS40. This construct and the p5 × GAL-Reporter vector were introduced into *Arabidopsis* protoplasts. A ProteoPrep® Total Extraction Sample Kit (Sigma) was employed to isolate total protein of *Arabidopsis* protoplasts according to the operating instructions of the kit, then the fluorescence activity of proteins was analyzed using the enzyme-labeled instrument. The LUC/REN ratio was used to measure the transcriptional activation of *JcMADS40*.

### Gene cloning and plant transformation

The coding sequence of *JcMADS40* was cloned by RT-PCR technology using the total RNA of physic nut seeds as a template with the *JcMADS40*-F and *JcMADS40*-R primers given in Additional File 5, and cloned into the pMD18-T vector. The correct coding sequence of *JcMADS40* gene was confirmed by sequencing. And then the coding sequence was excised from the pMD18-T vector after digestion with *Kpn* I and *Xba* I, then cloned into the pCAMBIA1301 vector at the *Kpn* I/*Xba* I site under the control of the CaMV 35S promoter. The constructed plant expression vectors were transferred into *Agrobacterium* (strain EHA105) by the freeze–thaw procedure, and strains containing this vectors were transferred into calli of rice cv. ZH11, according to the method of Tang [49].

### Phenotypic analysis and evaluation of the yield-related traits

Thirty individual plants of each of the *JcMADS40*-overexpressing (OE1, OE2, and OE3) and wild-type lines were employed to measure root and shoot lengths, 1000-grain weight, grain length and width, and yield per plant and to examine flower structure. Each line contained three independent biological replicates.

### RNA isolated and qRT-PCR analysis

Leaves from 2-week-old wild type and transgenic rice seedlings were collected and stored in ultra-low temperature refrigerator (− 80 °C) until required for use. Total RNA from different organs was isolated using a HiPure Plant RNA Mini Kit (Code No.R4151–02, Magen, http://www.magentec.com.cn/about.php). The cDNA synthesized using a PrimeScript™ IV 1st strand cDNA Synthesis Mix (TAKARA, Beijing, China). qRT-PCR was performed on a Mini Option real-time PCR system (LightCycler 480). Cycling conditions were performed according to the following parameters: 30 s at 95 °C, 5 s at 95 °C, 20 s at 60 °C, and 20 s at 72 °C. The reaction was carried out for 40 cycles. We used the 2^-∆∆CT^ method to detect relative transcript abundance, and rice *OsUbiquitin* gene was employed for normalization. The primers used employed in Additional File 5.

### Statistical analysis

In the research, each experiment contained three biological replicates. Statistical analysis was performed using SAS software package according to the Duncan multiple range test [50].

## Supplementary information

**Additional file 1 **Summary details of 63 *JcMADS* genes encoding MADS-domain proteins in physic nut.

**Additional file 2 **Summary of JcMADS proteins, and the reported biological functions of their orthologs in *Arabidopsis* and rice.

**Additional file 3.** The colored box represents the amino acid sequence of each conserved motif from each protein.

**Additional file 4 **Levels of expression of the 63 *JcMADS* genes in the organs detected (root, stem cortex, leaf, and seeds) according to RNA-seq data.

**Additional file 5.** Primers used in this study.

## Data Availability

Raw data about our research have been uploaded to NCBI’s SRA (sequence read archive) (accession nos. Are PRJNA257901 (for the drought stress data) and PRJNA244896 (for the salt stress data)). All JcMADS protein sequences obtained from our research are available at DDBJ/EMBL/GenBank (accession no. AFEW00000000NCBI). Our research related to other relevant data can be found in supplementary information files and published article.

## Abbreviations

HD: Homeodomain
ORF: Open reading frame
CDS: Coding sequence
