# Supplementary figures and images for: Genome-wide analysis of Jatropha curcas MADS-box gene family and functional characterization of the JcMADS40 gene in transgenic rice
Source: BMC Genomics. 2020 Apr 28;21:325. doi: 10.1186/s12864-020-6741-7 (PMC7187513; doi:10.1186/s12864-020-6741-7)

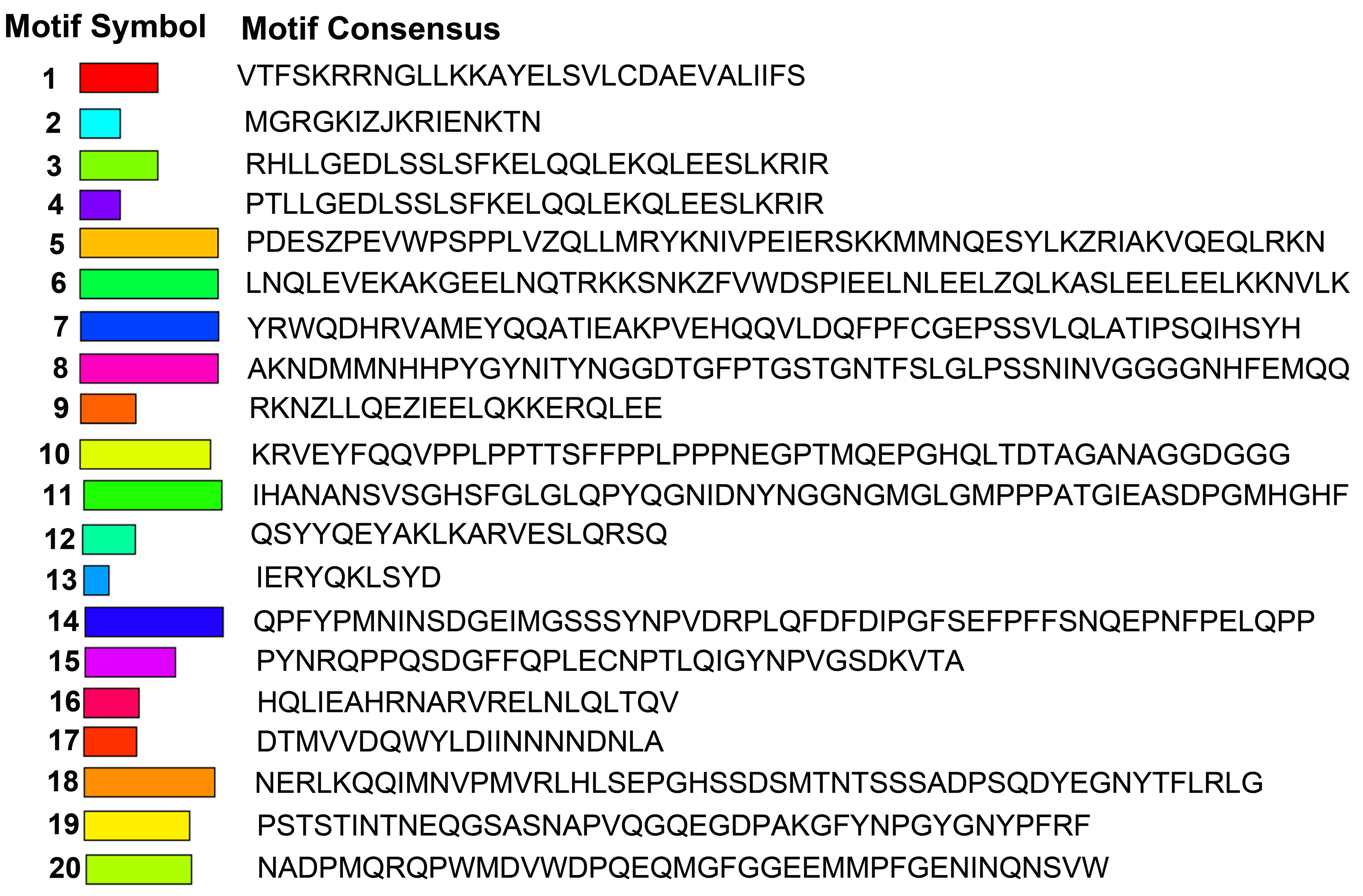

Supplement: Supplementary file 3 — Additional file 3. The colored box represents the amino acid sequence of each conserved motif from each protein. [file 12864_2020_6741_MOESM3_ESM.tif]
